# Anatomical Brain Changes and Cognitive Abilities in Patients with Obstructive Sleep Apnea Syndrome and Nonalcoholic Fatty Liver Disease

**DOI:** 10.1155/2021/8873652

**Published:** 2021-10-20

**Authors:** Branka Filipovic, Vesna Đuric, Natasa Filipovic, Stanimir Kiurski, Jamal Al Kiswani, Branka Markovic, Darko Laketic, Marija Marjanovic-Haljilji, Slobodan Kapor, Branislav R. Filipovic

**Affiliations:** ^1^University of Belgrade, Faculty of Medicine, Dr Subotica Starijeg 8, 11000 Belgrade, Serbia; ^2^Department of Gastroenterology, Clinical and Hospital Center “Dr Dragisa Misovic–Dedinje”, Heroja Milana Tepica 1, 11020 Belgrade, Serbia; ^3^University of Belgrade, Faculty of Sports and Physical Education, Blagoja Parovića 156, 11030 Belgrade, Serbia; ^4^Institute of Anatomy “Niko Miljanic”, Dr Subotica Starijeg 4/2, 11000 Belgrade, Serbia

## Abstract

Obstructive sleep apnea (OSA) is characterized by repetitive complete or partial collapse of the upper airway and reduction of airflow during sleep. It is associated with significantly increased daytime muscle sympathetic nerve activity thought to result from the repetitive intermittent periods of hypoxemia during sleep and brain alterations that are likely to result. Different brain regions are affected by subsequent hypoxia/anoxia. Neurodegenerative processes result in measurable atrophy of cortical gray matter in the temporal lobes and posterior cingulate cortex, as well as in subcortical structures such as the hippocampus, amygdala, and thalamus. This study involved a group of firstly diagnosed, therapy-naive, nonalcoholic fatty liver disease (NAFLD) patients, out of which 144 (96 males and 48 females), aged 34–57 (mean 47.88 ± 6.07), satisfied the recruiting criteria for the study and control groups. All the patients underwent MRI scanning, polysomnography testing, and cognitive evaluation. Cognitively, worse results were obtained in the group with OSA (*p* < 0.05) and NAFLD (*p*=0.047). A significant decrease in volumes of cortical and subcortical structures was revealed (*p* < 0.001). In conclusion, brain deterioration followed by cognitive impairment is, most likely, the result of intermittent hypoxia and anoxia episodes that initiate the domino process of deteriorating biochemical reactions in the brain.

## 1. Introduction

Obstructive sleep apnea (OSA) is characterized by repetitive complete or partial collapse of the upper airway and reduction of airflow during sleep. It is associated with significantly increased daytime muscle sympathetic nerve activity thought to result from the repetitive intermittent periods of hypoxemia during sleep and brain alterations that are likely to result [1, 2]. Some studies based on functional magnetic resonance imaging revealed attenuated signals in numerous regions of the brain: in the prefrontal, cingulate, and precuneus cortices; in the retrosplenial cortex, caudate nucleus, hippocampus, and parahippocampal regions; and within the dorsolateral pons, rostral ventrolateral medulla, medullary raphe, and midbrain [3, 4].

Studies conducted in younger and middle-aged adults indicate that the effects of sleep fragmentation and nocturnal hypoxemia probably support the cognitive deficits associated with OSA [5]. Sleep disruption with the compounding effect of hypoxemia could have deteriorating effects on brain integrity and morphology [6]. A wide range of cerebral gray matter changes has been associated with OSA, including cortical or volume changes across the temporal lobe and prefrontal cortex, and subcortical structures involving the hippocampus, thalamus, and cerebellum [7, 8].

Nonetheless, a relative paucity of investigations dealing with the interrelationships between OSA, brain integrity, and cognitive decline in older adults should be noted. As adults age, they may experience neurodegenerative processes resulting in measurable atrophy of cortical gray matter in the temporal lobes and posterior cingulate cortex, as well as subcortical structures such as the hippocampus, amygdala, and thalamus. These changes are evident even in the transitional or “at-risk” stages between normal aging and dementia, defined as those with subjective memory concerns and mild cognitive impairment [9–12].

A study by Elliott et al. [13] examined the cognitive symptoms of nonalcoholic fatty liver disease (NAFLD). They observed that increased cognitive difficulties were associated independently with functional difficulties in the NAFLD group compared with a healthy control group. A large cross-sectional study performed by Seo et al. [14] examined the association between NAFLD and cognitive impairment using computer-based tests of attention and reaction times.

They estimated that NAFLD was associated independently with reduced cognitive performance independent of cardiovascular disease and its risk factors. Previous studies on animals have shown a very strong connection between OSA and NAFLD [15]. Mesarwi et al. [15] showed that animals exposed to intermittent hypoxia (IH) have elevated blood pressure and develop sympathetic overactivation, atherosclerosis, and glucose as well as lipid dysregulation. OSA causes IH by recurrent collapse of the upper airway during sleep, as measured by peripheral oxyhemoglobin saturation. It may seem intuitive that arterial desaturation would result in intermittent tissue hypoxia as well.

However, no study has examined the tissue-specific effects of recurrent airway closure in humans. A few studies have shown that liver enzymes may be acutely elevated in OSA and are lowered with CPAP [16], and at least one study has shown that serum creatine phosphokinase similarly may be elevated in OSA and reduced by CPAP [17].

We aimed to assess whether OSA is associated with structural brain changes in various brain regions and whether OSA consecutively leads to cognitive impairment compared to NAFLD patients. Our goal was to compare the differences in cognitive functions in individuals with OSA and NAFLD relative to those with NAFLD but without OSA. We hypothesized that significant differences in cognitive statuses exist between those two groups of interest.

### 1.1. Materials and Methodology

This study involved a group of firstly diagnosed, therapy-naive NAFLD patients, out of which 144 (96 males and 48 females), aged 34–57 (mean 47.88 ± 6.07), satisfied recruiting criteria for the study and control groups. The grouping criterion for the division into the studied and control group was the presence of the OSA, so the studied group included the patients with NAFLD and OSA, and the controls were the individuals with NAFLD but without OSA. The study was approved by the Ethical Committee of the Clinical and Hospital Center “Dr Dragisa Misovic–Dedinje,” Belgrade.

All the participants were acquainted in detail with the study aim and design before entering the program. They all signed a written consent afterward.

**Figure 1:**
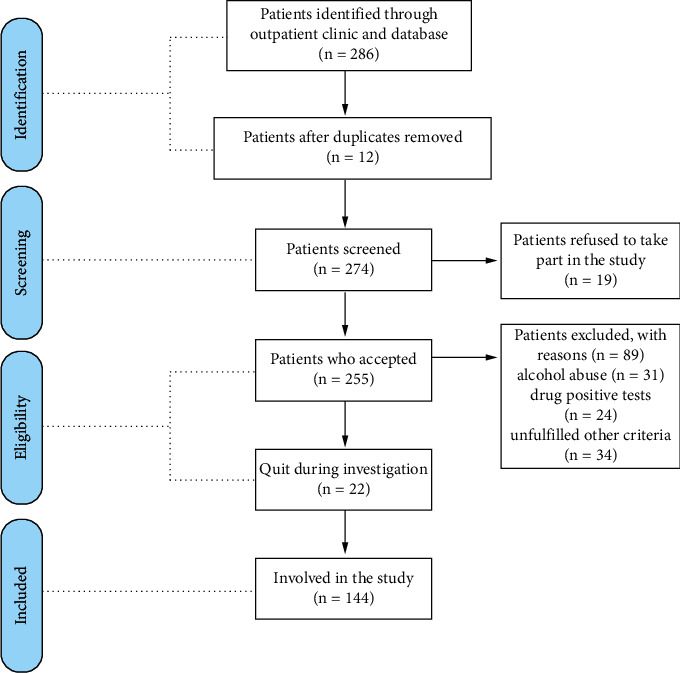
Patient selection flowchart.

A selection flow diagram is shown in Figure 1.

Recruiting criteria were as follows:

1. Older than 18.
2. No previous history of viral hepatitis of any kind, haemochromatosis, autoimmune hepatitis, cirrhosis, or other chronic liver diseases.
3. No presence of severe cardiopulmonary disease.
4. The absence of endocrinological disorders: hypothyroidism, hypercorticism, and syndrome of the polycystic ovaries.
5. No history or clinical signs of excessive alcohol abuse (>20 g/day for males and >10 g/day for females).
6. No neuropsychiatric disease involving signs of any kind of dementia, and/or neuropsychiatric medication history, or any other hepatotoxic drugs.
7. No visible traces of illicit drugs abuse. Negative urine multiple drug test on 10 kinds of drugs: cannabinoids, opiates, amphetamines, 3, 4-methylenedioxymethamphetamine, cocaine/crack, benzodiazepines, tricyclic antidepressants, barbiturates, methadone, and buprenorphine.
8. No visible focal or diffuse changes in the gray matter of the brain on MRI.
9. Fazekas score 0 on MRI scan. Fazekas score is the estimated level of the white matter vascular changes and is the aftermath of the brain vessels' atherosclerotic changes.
10. Absence of any rheumatologic disease.
11. Patients who used antidiabetic drugs, insulin, antilipemic drugs, uricosuric drugs, steroids, and oral contraceptives were excluded from the study.

## 2. Volumetric Procedures

Volume measurements of the gray and white matter and lateral ventricles of the brain were performed on 3D T1-weighted MR images (Phillips Inc. Holland). Acquisition parameters were as follows: TR = 9.8 ms; TE = 4.6 ms; flip angle = 8; section thickness = 1.2 mm; number of sections = 120; no section gap; whole-brain coverage; FOV = 224 mm; matrix = 192; reconstruction matrix = 256. Routine T2-weighted and FLAIR images were performed to rule out a mass lesion as a contributory factor to memory loss or cognitive decline. The structures were manually outlined and compared with automatic extraction of the regions of interest in commercially available software. The software finally computed the volumes required.

### 2.1. Cognitive Testing

After the diagnostic procedures, all the subjects underwent psychological testing of cognitive impairments using the Montreal Cognitive Assessment (MoCA) test, Serbian version. The test has several levels of testing: alternating connection (connect Figure 1 with letter A, then A to 2, to B, etc.), visuoconstructive abilities (draw a cube and a clock in 11:10 position of clock hands), memory (repeating numbers in the same and reverse order), attention (tap whenever you hear a letter A), serial subtraction of 7, starting with hundred, 100 − 7 = 93, 93 − 7 = 86, etc.), sentence repeating, and verbal fluency. The maximal score is 30, 26 being the threshold for normal cognitive functioning [18].

### 2.2. Laboratory Analysis

For body weight and height, the patients were measured in bare feet and light clothing in the morning with the same equipment. Body mass index (BMI) was calculated by dividing body weight by height square (kg/m^2^).

Fasting blood was taken in the morning for the measurement of serum glucose, and lipid profile comprising total cholesterol (TC), high-density lipoprotein-cholesterol (HDL-C), low-density lipoprotein-cholesterol (LDL-C), and triglycerides (TG). Adipokines, adiponectin, and leptin were analyzed and compared. All the tests were run by AO-BK-200 mini Auto Biochemistry Analyzer, Alpha Omega Electronics, Madrid, Spain.

### 2.3. Polysomnography

All participants, examined and controls, underwent polysomnography (PSG) in the sleep department. It was performed within four weeks of the MRI scan and neuropsychological testing. Nocturnal PSGs were collected on an ambulatory recording system with the Alice PDx portable diagnostic recording device (Philips Respironics), together with nasal airflow which was recorded with the nasal pressure transducer. Respiratory effort was assessed using thoracic and abdominal bands; blood oxygen saturation was revealed by pulse oximetry. Patients were advised not to disturb their usual bedtime weekly rhythm and were required to abstain from caffeinated beverages (coffee, caffeinated soda) at least eight hours before and, especially, during PSG data collection. The study was reported by an accredited sleep physician.

Sleep staging was scored according to the criteria of the American Academy of Sleep Medicine [19]. Apnea was defined as decrements in airflow ≥90% from baseline for ≥10 s. Hypopnea was defined as a 30% or greater decrease in flow lasting for ≥10 s and was associated with a 4% or greater oxyhemoglobin desaturation. The numbers of apneas and hypopneas per hour of sleep were calculated to obtain the apnea-hypopnea index (AHI). The oxygen desaturation index (ODI) was defined as the number of dips in oxygen saturation (SpO_2_) ≥4% per hour of total sleep time. OSA was defined as normal: AHI < 5; mild sleep apnea: 5 ≤ AHI < 15; moderate sleep apnea: 15 ≤ AHI < 30; and severe sleep apnea: AHI ≥ 30 events/h.

The atherogenic index of plasma (AIP) represents the risk for atherosclerosis. It is calculated as the logarithmic value of the triglyceride/HDL score. The risk was interpreted as follows: AIP < 0.11 low risk; AIP = 0.11–0.21 intermediate risk; and API > 0.21 increased risk. The following index values were analyzed: cholesterol/HDL, triglycerides/HDL, and HDL/LDL.

### 2.4. Ultrasonography Evaluation

The liver was assessed as normal when the consistency was homogeneous, displayed fine level echoes, minimally hyperechoic or even isoechoic in contrast to the regular renal cortex. Mild steatosis was evaluated as a minor increase in liver echogenicity. In moderate steatosis, there were visual images associated with intrahepatic vessels, the slightly damaged diaphragm, and the existence of increased liver organ echogenicity. Severe steatosis was evaluated as a marked increase in hepatic echogenicity, poor penetration of posterior segment from the right lobe of the liver, and poor or no visual images from the hepatic vessels and the diaphragm [20].

FibroScan® (Echosens group) was used to determine the fibrosis grade in the liver parenchyma. The normal range for a FibroScan is between 2 and 7 kPa. The average normal result is 5.3 kPa. The results vary based on the liver disease in question. For NASH/NAFLD there are 4 stages of scarring: • *F*_0_ to *F*_1_ means no scarring or mild fibrosis, 2–7 kPa; • *F*_2_ is moderate fibrosis, 7–10 kPa; • *F*_3_ is severe fibrosis, 10–14 kPa; and • *F*_4_ is cirrhosis or advanced fibrosis higher than 14 kPa.

### 2.5. Statistical Evaluation

Statistical testing was performed by the commercially available software (SPSS 17.0, Inc., Chicago Il, US). Besides measures of the central tendency (mean and standard deviation (SD), minimum and maximum), potential differences of mean values were assessed with one-way analysis of variance (ANOVA) with the Bonferroni post hoc correction, Student's *t*-test for independent samples for parametric, and the chi-squared test for nonparametric data. The correlation between the variables was estimated using Spearman and Pearson's correlation coefficient. Multivariate linear regression was performed with volumes of brain structures as a dependent variable, while OSA and NAFLD degrees were independent predictors, adjusting for gender, age, BMI, cholesterol level, adiponectin, and leptin. Statistical hypotheses were analyzed at the level of significance of 0.05.

## 3. Results

### 3.1. Demographic Data

Demographic data are shown in Table 1. Males dominated in the groups with sleep apnea. The body mass index was significantly higher among the persons with OSA.

### 3.2. Laboratory Results

The concentration of serum triglyceride, HDL, and LDL differed significantly: patients with severe OSA had the lowest concentration of HDL and the highest level of LDL. All the examinees with OSA were at very high risk for atherosclerosis (all above 0.51 risk index).

### 3.3. Brain Volumes Changes

Although total brain volumes among the groups observed did not differ significantly, volumes of the structures of interest were significantly lower in the group of examinees with OSA. Higher volumes were obtained for the lateral ventricles on both hemispheres in OSA suffering patients, while volumes of the amygdaloid complexes did not differ significantly (Table 2).

### 3.4. Association between Liver Steatosis and Fibrosis and OSA

The severity of OSA differed among the observed groups with liver steatosis (Table 3). The patients with serious steatosis were numerous among those with severe OSA. The severity of NAFLD is associated with the increase in OSA severity (rho = 0.214; *p*=0.010). Level of fibrosis estimated with the Fibro scan correlated with the grade of steatosis (*B* = 0.56, beta = 0.73, *t* = 13.09, significance *p* < 0.0001), as well as with the severity of OSA (*B* = 0.89, beta = 0.13, *t* = 2.37, significance *p*=0.019).

### 3.5. Level of Cognitive Deficit

According to the MoCA score, the groups divided by the grade of liver steatosis differed (*F* = 2.72, DF = 3, 140, *p*=0.047).

Regarding the severity of OSA, the level of the cognitive deficit did not differ among the obtained groups.

Discriminative function analysis outlined cognitive level as the only parameter of importance for the classification of a newly obtained patient into one of the groups of interest: equation = −9.19 + 0.37 × MoCA; centroids for groups: mild, −0.17; moderate, −0.63; severe, 0.25 and cutoff points: mild to moderate, −0.23; moderate to severe, −0.195; goodness of classification, 71.50%.

### 3.6. Polysomnography Results

Polysomnographic parameters had an inverse influence on the volumes of the structures of interest. The tested subjects had lower volumes when both AHI and ODI were higher (Table 4). Atherogenic index of plasma correlated with AHI (*r*_2_ = 0.48, constant = 17.63, *b*_1_ = 12.27, *p*=0.015), ODI (*r*_2_ = 0.75 constant = 16.182, *b*_1_ = 14.29 *p*=0.02), and BMI (*r*_2_ = 0.106, constant = 28.17, *b*_1_ = 5.45, *p* < 0.001).

### 3.7. Brain Volumes in Patients with OSA

In the multivariate regression analyses the patients with higher levels OSA showed a significant reduction in all volumes of brain structures except for amygdaloid complex and white matter volume (Table 5).

## 4. Discussion

The study aimed to reveal whether OSA is associated with structural brain changes in diverse brain regions and whether the grade of liver steatosis influences both OSA appearance and subsequent cognitive alterations, as the result of IH.

Our results indicate that the volume changes of the overall cortex and basal nuclei are related to AHI and ODI as the main parameters and both strongly influenced volume decrease. These results are in correlation with previous studies which showed changes in volume values of the brain structures of interest. Kim et al. [21] investigated the effect of long-term treatment on brain volume in patients with OSA and their results have shown a significant increase in volume in the medial prefrontal cortex, superior frontal cortex, precuneus, and posterior temporal cortex, as well as in the dentate gyrus of hippocampus, thalamus, and cerebellum including the dentate nucleus. Fatouleh et al. [3] conducted a study on patients with OSA and their results showed significant changes in volume in the left and right parts of the insula, dorsolateral prefrontal cortex, dorsal precuneus, sensorimotor cortex, and posterior temporal cortex, as well as anterior cingulate cortex, retrosplenial cortex, and caudate nucleus. All mentioned brain structures, previously hippocampus and medial prefrontal cortex, are involved in the regulation of sleep and have direct anatomical connections with the pre-Bötzinger Complex (preBötC), a compact medullary region essential for generating normal breathing rhythm and pattern [22].

OSA causes nocturnal intermittent hypoxemia and sleep fragmentation in response to oxygen desaturation. Some investigators indicated that OSA, vascular depression, and cognitive impairment are linked to several pathologic processes in the cerebral microvascular and neurovascular systems [23, 24]. In OSA repetitive episodes of the intracranial blood flow, an unexpected increase during apneic episodes caused damage to the endothelial cells of small arteries and arterioles, which result in decreased endothelial vasodilator production such as nitric oxide. Moreover, IH during sleep in patients with OSA can contribute to apoptosis and atrophy within the hippocampal structure, resulting in learning, mnemonic, attentional, and executive function deficits [24]. Filipovic et al. [25] investigated the possible correlation between cognitive status and NAFLD using the MoCA test, finding a lower MoCA score and a reduction in white and gray brain volumes in NAFLD patients. The patients with NAFLD have a risk four times higher than manifesting lower cognitive abilities and depleted cognitive performance and deficit.

The major risk factors for OSA include obesity, male sex, alcohol and smoking habits, a family history of OSA, and upper airway structural abnormalities such as a large neck girth and craniofacial abnormalities. NAFLD is most commonly associated with metabolic risk factors, such as obesity, diabetes mellitus type 2, and elevated triglyceride levels, but some recent studies have reported that chronic intermittent hypoxia (CIH) can be an independent risk factor to induce liver damage [26, 27]. OSA and episodes of repetitive IH induce insulin resistance and dyslipidemia which are involved in NAFLD pathogenesis. CIH increases the expression of the hypoxia-inducible factor 1-alpha and that of downstream genes involved in lipogenesis, increasing *β*-oxidation and, consequently, leading to exacerbation of oxidative stress in the liver. OSA also disrupts the gut-liver axis, increasing intestinal permeability with a possible role of gut microbiota in the link between OSA and NAFLD [28].

Several studies examined OSA influence on structural changes using magnetic resonance spectroscopy, the decreased N-acetyl aspartate, and choline concentration in prefrontal subcortical white matter. Alterations revealed the early structural changes such as neuronal loss and axonal damage [29–31].

In some magnetic resonance spectroscopy-guided studies, the left hippocampus was stressed as a region especially sensitive to intermittent hypoxia in OSA suffering patients [32]. Our results oppose the findings from the study of Yaouhi et al. [8] who reported a large variety of structures susceptible to OSA: bilateral inferior gyri, right temporal cortex, occipital cortex, right thalamus, left caudate nucleus and left globus pallidus, right hippocampal gyrus, cerebellar hemisphere on the same side, and vermis. Morrell et al. [33] reported changes in the left temporal lobe and right cerebellar hemisphere. Joo et al. [34] also pointed out a significant volume decrease in the left straight gyrus anterior cingulate cortex, the right insular cortex, caudate nucleus, amygdala–hippocampus complex, the inferior temporal gyrus, and cerebellum, in OSA patients versus healthy controls. The study performed by Torelli et al. [35] indicated the changes in bilateral caudate nucleus volume, but the change was relative and dependent on the presence of hypertension and cigarette smoking. If those two factors were checked for analysis, there would be no difference between the tested and control group. Cholesterol concentration differences were insignificant in our study, although the cholesterol/HDL ratio significantly differed between OSA patients and the controls. HDL concentrations showed higher values in the control group, while LDL was significantly higher in the group with severe OSA.

The atherogenic index of plasma was significantly lower in the controls but correlated with AHI, ODI, and BMI indexes. On the contrary, Sparks et al. [36] claimed that cholesterol concentrations may influence poorer cognitive performance. This disproportion may be related to the usage of different cognitive tests: in our study, the MoCA test was used as the main testing questionnaire, but Sparks and his team applied Mini-Mental State Examination for the evaluation of the cognitive state. According to the opinion of Rademeyer et al. [37], MoCA is a more potential screening tool for cognitive impairment. In the meta-analysis provided by Siqueira et al. [38], thirty-seven studies suggested that MoCA is a more sensitive tool for neurocognitive disorders detection because it assesses executive function and visuospatial abilities. Finally, the discriminative analysis function, according to our results, outlined the MoCA score as the only parameter of importance for the classification of a newly obtained subject into one of the groups of interest, selected by the OSA severity, with almost 80% of accuracy.

An et al. [39] stated that the higher intake of cholesterol negatively influences the cognitive state of the middle age Chinese population. Generally, lower values of HDL are indicated for the poorer cognitive state, or inversely, higher concentrations of HDL are indicators of cognitive improvement. The atherogenic index of plasma is nowadays considered a novel predictor of NAFLD, and NAFLD itself negatively influences the cognitive status as previously reported [40].

The role of two adipokines, leptin and adiponectin, in cognitive regulation is at least equivocal and insufficiently elucidated, considering literature data. Our patients with OSA had lower concentrations of adiponectin, but this difference was not statistically verified. It has already been published that adiponectin is lower in the examinees with OSA [41, 42], and OSA cohabitates with depression symptoms [43]. The patients with NAFLD are prone to depression [36]; furthermore, the depression appearance is related to the white matter loss which corresponds to the liver fibrosis grade [44].

Leptin values in our study are higher in patients with OSA and NAFLD, compared to the controls with NAFLD only. The highest values were noted in persons with severe OSA. Leptin is a potent ventilation stimulant acting on central respiratory control nuclei. The central satiety effects of leptin are abrogated in obesity. Leptin resistance is defined as a failure of high-circulating levels of leptin to decrease hunger and promote energy expenditure.

OSA and IH, powerful triggers of oxidative stress, increase peripheral leptin levels and also induce leptin resistance (for a detailed review, see [45]).

The statistical trend for lower leptin levels to be associated with higher cognitive scores was revealed in a large sample of 2731 subjects measured by MoCA. Excessive leptin per unit of fat was associated with lower total MoCA score and memory in black men and with higher MoCA scores in white men [46]. In our investigation, there was no possibility to explore different races because our examinees were strictly Caucasians. Whether this finding indicates that leptin has a different role in various anthropological types remains yet to be examined.

In our study, two indexes, characteristic for OSA, AHI, and ODI, were found to have a strong influence on the reduction of the volumes of almost all regions of interest. Only amygdaloid complexes were spared from the volume reduction. Total brain volume was found insignificantly different in our sample, which could be the result of larger lateral ventricle volumes and, subsequently, possible higher quantities of cerebrospinal liquor. OSA, that is, impaired AHI and ODI indexes, likely causes cognitive impairment through IH, hormonal imbalance, and/or systemic inflammation, either independently or via the resultant endothelial dysfunction that occurs. Still, the cognitive defect is only partially reparable after CPAP treatment (for a detailed review, see [47]). In most of the studies obtained, the hippocampal reduction is related to poorer oxygen saturation and higher number of apnea–hypopnea episodes, or poorer blood flow [7, 48]. The influence of OSA on the basal ganglia, particularly on striatal components (caudate nucleus, putamen, and globus pallidus) reduction, shows executive dysfunction, cognitive slowing, working memory deficits, attentional dysfunction, memory retrieval difficulty, impaired language, disturbances (depression, anxiety, and irritability), and impaired procedural memory (for a detailed review, see [49]). The prefrontal cortex is susceptible to hypoxia according to the fMRI-guided study, as indicated by Zhang et al. [50]. Besides prefrontal, lower blood flow was gained in the anterior cingulate cortex, a part with multiple, but foremost emotion-related functions.

CPAP is the first-line treatment for NAFLD patients with OSA, but the effect of CPAP treatment on liver disease is still controversial and unclear. CPAP treatment may be beneficial to NAFLD patients with OSA independent of metabolic risk factors, but a sufficiently long therapeutic duration longer than three months may be needed to achieve positive effects on the liver enzymes and liver steatosis especially in patients with moderate-to-severe OSA [26, 51]. These data also suggest that CPAP can prevent the progression of NAFLD in OSA individuals. Ng et al. [52] detected significant correlations between hepatic steatosis and markers of severity of OSA but did not show that CPAP alone improves liver steatosis and fibrosis. Weight reduction in obese NAFLD individuals with OSA is associated with an improvement in OSA severity and reduced upper airway collapsibility. Therefore, further research is needed regarding the impact of weight loss and changes in lifestyle and dietary habits on the improvement of liver steatosis and fibrosis in patients with OSA.

The limitations of this study are as follows:

1. This is a referral, not a cohort study, restricted only to the patients referred to our department and outpatient clinics
2. A relatively small number of patients with mild and moderate OSA were investigated
3. Only noninvasive tests were performed for NAFLD
4. It is limited to newly diagnosed, therapy-naive patients
5. The diagnoses were dependable on ultrasonographer and sleep doctor's skills and experience
6. There was an inability to perform functional magnetic resonance imaging because our institution does not possess one

## 5. Conclusion

Syndrome of OSA worsens the cognitive status in patients with NAFLD. The possible underlying mechanism is the influence on the reduction of cortical and subcortical structures driven by constant apnea/hypopnea episodes, and consecutive hypoxia that initiates the domino process of deteriorating biochemical reactions in the brain.

## Figures and Tables

**Table 1:** Demographic parameters of examined population.

Group according to sleep apnea severity	Examined	Controls	Total	Significance
	(*N* = 68)	(*N* = 76)	(*N* = 144)	
Parameter				
Age (years ± SD)	47.88 ± 6.07	47.62 ± 6.97	46.94 ± 9.00	NS
Gender (male/female)	58/10	38/38	96/48	*p* < 0.001
Education level (grammar/high school/university)	3/7/12	25/33/18	43/70/31	NS
Body mass index (BMI, kg/m^2^)	35.34 ± 7.31	34.95 ± 8.27	31.90 ± 6.61	*F* = 807.33, DF = 3.140, *p* < 0.001
Leptin (ng/mL) m/f	11.36 ± 1.97 22.78 ± 3.28	4.39 ± 2.17 11.44 ± 3.23	10.39 ± 2.53 14.55 ± 3.11	*F* = 5.66, DF = 3.140, *p* < 0.001
Adiponectin (ng/mL) m/f	8.06 ± 0.97 11.14 ± 2.03	8.8 ± 1.44 13.71 ± 3.63	8.19 ± 2.44 11.43 ± 3.69	NS
Glucose (mmol/l)	5.31 ± 0.65	5.75 ± 1.55	5.52 ± 1.28	NS
C reactive protein (mg/l, mean ± SE)	3.86 ± 1.27	3.74 ± 0.39	3.39 ± 0.25	NS
Cholesterol (mmol/l, mean ± SD)	5.19 ± 0.63	5.74 ± 1.18	5.57 ± 1.11	NS
HDL (mmol/l, mean ± SE)	0.95 ± 0.04	1.37 ± 0.07	1.18 ± 0.04	*F* = 8.94, DF = 3.140, *p* < 0.001
LDL (mmol/l, mean ± SE)	3.23 ± 0.17	3.27 ± 0.12	3.44 ± 0.08	*F* = 2.76, DF = 3.140, *p* < 0.05
Triglycerides (mmol/l, mean ± SE)	2.68 ± 0.35	1.86 ± 0.13	2.24 ± 0.12	*F* = 7.64, DF = 3.140, *p* = 0.009
Cholesterol/HDL ratio (mean ± SE)	6.02 ± 0.33	4.64 ± 0.18	5.27 ± 0.18	*F* = 8.035, DF = 3.140, *p* < 0.001
Triglycerides/HDL ratio	3.32 ± 0.48	1.62 ± 0.15	2.41 ± 0.19	*F* = 7.71, DF = 3.140, *p* < 0.0001
AIP	0.52	0.21	0.38	*F* = 8.01, DF = 3.140, *p* < 0.001
HDL/LDL ratio (mean ± SE)	0.32 ± 0.03	0.43 ± 0.04	0.38 ± 0.02	NS
AHI per hour (mean ± SE)	12.23 ± 5.49	2.95 ± 0.12	12.16 ± 1.31	*F* = 29.44, DF = 3>140, *p* < 0.001
ODI per hour (mean ± SE)	14.35 ± 4.51	2.78 ± 0.13	11.08 ± 1.24	*F* = 25.64, DF = 3.140, *p* < 0.001
Total brain volume (cm^3^, mean ± SE)	1441.19 ± 32.31	1403.37 ± 18.85	1398.96 ± 13.16	NS
MoCA score (mean ± SD)	24.23 ± 3.14	25.53 ± 3.20	25.28 ± 3.04	*F* = 2.72, DF = 3.140, *p* < 0.05

NS: not significant.

**Table 2:** Brain volumes according to the sleep apnea of the observed groups.

Group according to sleep apnea severity	Mild (*N* = 11)		Moderate (*N* = 14)		Severe (*N* = 43)		Controls (*N* = 76)		Total (*N* = 144)		Significance	
	L	R	L	R	L	R	L	R	L	R	L	R
Caudate nucleus volume (cm^3^, mean ± SD, L/R)	3.76 ± 0.11	3.80 ± 0.15	3.77 ± 0.04	3.81 ± 0.16	3.79 ± 0.15	3.85 ± 0.14	4.5 ± 0.65	4.56 ± 0.64	4.22 ± 0.6	4.17 ± 0.6	*F* 3.140 = 26.33, *p* < 0.001	*F* 3.140 = 27.93, *p* < 0.001
Putamen volume (cm^3^, mean ± SD, L/R)	5.94 ± 0.10	5.95 ± 0.10	5.97 ± 0.11	5.98 ± 0.11	5.97 ± 0.12	5.99 ± 0.12	6.31 ± 0.62	6.30 ± 0.70	6.15 ± 0.49	6.16 ± 0.54	*F* 3.140 = 6.51, *p* < 0.001	*F* 3.140 = 5.03, *p*=0.002
Globus pallidus volume (cm^3^, mean ± SD, L/R)	2.13 ± 0.10	2.12 ± 007	2.13 ± 0.08	2.61 ± 0.46	2.18 ± 0.79	2.12 ± 0.07	2.61 ± 0.46	2.58 ± 0.40	2.38 ± 0.41	2.36 ± 0.37	*F* 3.140 = 24.20, *p* < 0.001	*F* 3.140 = 27.06, *p* < 0.001
Thalamus volume (cm^3^, mean ± SD, L/R)	6.29 ± 0.10	6.28 ± 0.09	6.27 ± 0.09	6.26 ± 0.09	6.28 ± 0.07	6.28 ± 0.07	7.46 ± 0.77	7.48 ± 0.90	6.90 ± 0.81	6.91 ± 0.79	*F* 3.140 = 52.12, *p* < 0.001	*F* 3.140 = 38.77, *p* < 0.001
Hippocampal formation volume (cm^3^, mean ± SD, L/R)	2.82 ± 0.12	2.81 ± 0.12	2.77 ± 0.13	2.77 ± 0.13	2.80 ± 0.13	2.80 ± 0.13	3.99 ± 0.63	3.96 ± 0.68	3.43 ± 0.75	3.41 ± 0.77	*F* 3.140 = 78.02, *p* < 0.001	*F* 3.140 = 63.94, *p* < 0.001
Lateral ventricle volume (cm^3^, mean ± SD, L/R)	7.48 ± 0.14	7.49 ± 0.14	7.49 ± 0.13	7.51 ± 0.13	7.50 ± 0.14	7.51 ± 0.14	5.86 ± 0.55	5.81 ± 0.54	6.63 ± 0.92	6.61 ± 0.94	*F* 3.140 = 183.72, *p* < 0.001	*F* 3.140 = 205.44, *p* < 0.001
Amygdaloid complex volume (cm^3^, mean ± SD, L/R)	1.65 ± 0.11	1.64 ± 0.12	1.61 ± 0.08	1.61 ± 0.08	1.67 ± 0.08	1.68 ± 0.08	1.68 ± 0.19	1.69 ± 0.21	1.67 ± 0.15	1.68 ± 0.17	NS	NS
Prefrontal cortex volume (cm^3^, mean ± SD, L/R)	140.45 ± 6.20	141.54 ± 6.07	143.43 ± 8.16	144.50 ± 8.05	145.93 ± 8.43	146.98 ± 8.36	161.16 ± 18.85	158.84 ± 19.32	153.30 ± 16.95	152.58 ± 16.46	*F* 3.140 = 15.41, *p* < 0.001	*F* 3.140 = 9.55, *p* < 0.001
Gray matter volume (cm^3^, mean ± SD)	351.91 ± 4.91		348.79 ± 6.02		348.93 ± 7.57		341.43 ± 40.03		335.79 ± 29.93		*F* 3.140 = 35.34, *p* < 0.001	

**Table 3:** Distribution of steatosis severity among groups with different levels of OSA.

OSA	Grade of liver steatosis			
	Mild	Moderate	Severe	Total
Mild	11	0	0	11
Moderate	2	8	4	14
Severe	12	13	18	43
Without	36	25	15	76
Total	61	46	37	144

rho = 0.214; *p*=0.010.

**Table 4:** Correlation matrix between polysomnographic parameters and volumes obtained.

Parameter	AHI per hour		ODI per hour		Significance AHI		Significance ODI	
	L	R	L	R	L	R	L	R
Caudate nucleus volume (cm^3^)	*B* = −0.015 Const = 4.35 Beta = −0.37	*B* = −0.015 Const = 4.40 Beta = −0.39	*B* = −0.015 Const = 4.34 Beta = −0.38	*B* = −0.015 Const = 4.38 Beta = −0.38	*p* < 0.001	*p* < 0.001	*p* < 0.001	*p* < 0.001
Putamen volume (cm^3^)	*B* = −0.01 Const = 6.24 Beta = −0.25	*B* = −0.01 Const = 6.25 Beta = −0.22	*B* = −0.07 Const = 6.23 Beta = −0.22	*B* = −0.07 Const = 6.24 Beta = −0.198	*p* < 0.005	*p* < 0.01	*p* < 0.01	*p* < 0.02
Globus pallidus volume (cm^3^)	*B* = −0.01 Const = 2.47 Beta = −0.38	*B* = −0.01 Const = 2.45 Beta = −0.36	*B* = −0.01 Const = 2.49 Beta = −0.35	*B* = −0.09 Const = 2.46 Beta = −0.37	*p* < 0.0001	*p* < 0.0001	*p* < 0.0001	*p* < 0.0001
Thalamus volume (cm^3^)	*B* = −0.02 Const = 7.19 Beta = −0.45	*B* = −0.02 Const = 7.20 Beta = −0.42	*B* = −0.024 Const = 7.16 Beta = −0.43	*B* = −0.024 Const = 7.18 Beta = −0.40	*p* < 0.001	*p* < 0.0001	*p* < 0.0001	*p* < 0.0001
Hippocampal formation volume (cm^3^)	*B* = −0.02 Const = 3.72 Beta = −0.49	*B* = −0.02 Const = 3.69 Beta = −0.48	*B* = −0.02 Const = 3.69 Beta = −0.47	*B* = −0.02 Const = 3.69 Beta = −0.48	*p* < 0.0001	*p* < 0.0001	*p* < 0.0001	*p* < 0.0001
Lateral ventricle volume (cm^3^)	*B* = 0.03 Const = 6.25 Beta = 0.54	*B* = 0.03 Const = 6.21 Beta = 0.54	*B* = 0.03 Const = 6.25 Beta = 0.54	*B* = 0.03 Const = 6.22 Beta = 0.54	*p* < 0.0001	*p* < 0.0001	*p* < 0.001	*p* < 0.001
Prefrontal cortex volume (cm^3^)	*B* = −0.36 Const = 157.65 Beta = −0.33	*B* = −0.29 Const = 156.13 Beta = −0.28	*B* = −0.36 Const = 157.36 Beta = −0.32	*B* = −0.30 Const = 155.89 Beta = −0.27	*p* < 0.0001	*p* < 0.001	*p* < 0.001	*p* < 0.001
Gray matter volume (cm^3^)	*B* = −1.24 Const = 396.63 Beta = −0.42		*B* = −1.23 Const = 395.21 Beta = −0.39		*p* < 0.001		*p* < 0.001	

L: left hemisphere; R: right hemisphere.

**Table 5:** Results of the multivariate regression analyses.

Brain structures	Grade of OSA		Grade of liver steatosis	
	L	R	L	R
Caudate nucleus volume (cm^3^)	*B* = −0.256 *p* < 0.001	*B* = −0.258 *p* < 0.001	*B* = 0.042 *p*=0.443	*B* = 0.026 *p*=0.632
Putamen volumes (cm^3^)	*B* = −0.124 *p*=0.002	*B* = −0.125 *p*=0.001	*B* = 0.061 *p*=0.225	*B* = 0.083 *p*=0.144
Globus pallidus volume (cm^3^)	*B* = −0.173 *p* < 0.001	*B* = −0.171 *p* < 0.001	*B* = 0.092 *p*=0.013	*B* = 0.133 *p* < 0.001
Thalamus volume (cm^3^)	*B* = −0.407 *p* < 0.001	*B* = −0.419 *p* < 0.001	*B* = 0.007 *p* = 0.912	*B* = 0.044 *p* = 0.559
Hippocampal formation volume (cm^3^)	*B* = −0.415 *p* < 0.001	*B* = −0.406 *p* < 0.001	*B* = 0.003 *p*=0.951	*B* = −0.001 *p*=0.989
Lateral ventricle volume (cm^3^)	*B* = −0.014 *p*=0.422	*B* = −0.007 *p*=0.324	*B* = −0.047 *p*=0.393	*B* = 0.006 *p*=0.920
Amygdaloid complex volume (cm^3^)	*B* = −0.011 *p*=0.351	*B* = −0.009 *p*=0.461	*B* = 0.021 *p*=0.207	*B* = 0.019 *p*=0.293
Prefrontal cortex volume (cm^3^)	*B* = −5.527 *p* < 0.001	*B* = −4.366 *p* < 0.001	*B* = 0.309 *p*=0.853	*B* = 0.143 *p*=0.932
White matter volume (cm^3^)	*B* = −3.866 *p*=0.081		*B* = 1.017 *p*=0.745	
Gray matter volume (cm^3^)	*B* = −20.629 *p* < 0.001		*B* = 5.224 *p*=0.184	

## Data Availability

The data used to support the findings of this study are deposited in the DOI repository.

## References

[1] Fatouleh R., McKenzie D. K., Macefield V. G. (2014). Respiratory modulation of muscle sympathetic nerve activity in obstructive sleep apnoea. *Experimental Physiology*.

[2] Fatouleh R. H., Lundblad L. C., Macey P. M., McKenzie D. K., Henderson L. A., Macefield V. G. (2015). Reversal of functional changes in the brain associated with obstructive sleep apnoea following 6 months of CPAP. *NeuroImage: Clinical*.

[3] Fatouleh R. H., Hammam E., Lundblad L. C., McKenzie D. K., Henderson L. A., Macefield V. G. (2014). Functional and structural changes in the brain associated with the increase in muscle sympathetic nerve activity in obstructive sleep apnoea. *NeuroImage: Clinical*.

[4] Lundblad L. C., Fatouleh R. H., Hammam E., McKenzie D. K., Macefield V. G., Henderson L. A. (2014). Brainstem changes associated with increased muscle sympathetic drive in obstructive sleep apnoea. *NeuroImage*.

[5] Norton S., Matthews F. E., Barnes D. E., Yaffe K., Brayne C. (2014). Potential for primary prevention of Alzheimer’s disease: an analysis of population-based data. *The Lancet Neurology*.

[6] Macedo A. C., Balouch S., Tabet N. (2017). Is sleep disruption a risk factor for Alzheimer’s disease?. *Journal of Alzheimer’s Disease*.

[7] Canessa N., Castronovo V., Cappa S. F. (2011). Obstructive sleep apnea: brain structural changes and neurocognitive function before and after treatment. *American Journal of Respiratory and Critical Care Medicine*.

[8] Yaouhi K., Bertran F., Clochon P. (2009). A combined neuropsychological and brain study of obstructive sleep apnea. *Journal of Sleep Research*.

[9] Cross N. E., Memarian N., Duffy S. L. (2018). Structural brain correlates of obstructive apnoea in older adults at risk for dementia. *European Respiratory Journal*.

[10] Celikbilek A., Celikbilek M., Bozkurt G. (2018). Cognitive assessment of patients with nonalcoholic fatty liver disease. *European Journal of Gastroenterology and Hepatology*.

[11] Jockschat T., Kleiman A., Schulz J. B. (2012). Neuroanatomic changes and their association with cognitive decline in mild cognitive impairment: a meta-analysis. *Brain Structure and Function*.

[12] Ryu S. Y., Lim E. Y., Na S. (2017). Hippocampal and entorhinal structures in subjective memory impairment: a combined MRI volumetric and DTI study. *International Psychogeriatrics*.

[13] Elliott C., Frith J., Day C. P., Jones D. E., Newton J. L. (2013). Functional impairment in alcoholic liver disease and non-alcoholic fatty liver disease is significant and persists over 3 years of follow-up. *Digestive Diseases and Sciences*.

[14] Seo S. W., Gottesman R. F., Clark J. M. (2016). Nonalcoholic fatty liver disease is associated with cognitive function in adults. *Neurology*.

[15] Mesarwi O. A., Loomba R., Malhotra A. (2019). Obstructive sleep apnea, hypoxia, and nonalcoholic fatty liver disease. *American Journal of Respiratory and Critical Care Medicine*.

[16] Chen L. D., Lin L., Zhang L. J. (2018). Effect of continuous positive airway pressure on liver enzymes in obstructive sleep apnea: a meta-analysis. *The Clinical Respiratory Journal*.

[17] Bajantri B., Lvovsky D. (2018). A case of concomitant obstructive sleep apnea and non-alcoholic steatohepatitis treated with CPAP therapy. *Gastroenterology Research*.

[18] Kljajevic V. (2009). Montreal cognitive assessment–Serb’s version. Aktuelnosti iz neurologije. *Psihijatrije I Granicnih Podrucja*.

[19] Tapia I. E., Karamessinis L., Bandla P. (2008). Polysomnographic values in children undergoing puberty: pediatric vs. adult respiratory rules in adolescents. *Sleep*.

[20] De Lucia Rolfe E., Brage S., Sleigh A. (2018). Validity of ultrasonography to assess hepatic steatosis compared to magnetic resonance spectroscopy as a criterion method in older adults. *PLoS One*.

[21] Kim H., Joo E., Suh S., Kim J. H., Kim S. T., Hong S. B. (2016). Effects of long-term treatment on brain volume in patients with obstructive sleep apnea syndrome. *Human Brain Mapping*.

[22] Yang C. F., Feldman J. L. (2018). Efferent projections of excitatory and inhibitory preBötzinger Complex neurons. *Journal of Comparative Neurology*.

[23] Kerner N. A., Roose S. P. (2016). Obstructive sleep apnea is linked to depression and cognitive impairment: evidence and potential mechanisms. *American Journal of Geriatric Psychiatry*.

[24] Liu X., Ma Y., Ouyang R. (2020). The relationship between inflammation and neurocognitive dysfunction in obstructive sleep apnea syndrome. *Journal of Neuroinflammation*.

[25] Filipović B., Marković O., Đurić V., Filipović B. (2018). Cognitive changes and brain volume reduction in patients with nonalcoholic fatty liver disease. *Chinese Journal of Gastroenterology and Hepatology*.

[26] Liu X., Miao Y., Wu F., Du T., Zhang Q. (2018). Effect of CPAP therapy on liver disease in patients with OSA: a review. *Sleep and Breathing*.

[27] Lin Q. C., Chen L. D., Chen G. P. (2015). Association between nocturnal hypoxia and liver injury in the setting of nonalcoholic fatty liver disease. *Sleep and Breathing*.

[28] Aron-Wisnewsky J., Clement K., Pépin J. L. (2016). Nonalcoholic fatty liver disease and obstructive sleep apnea. *Metabolism*.

[29] Alchanatis M., Deligiorgis N., Zias N. (2004). Frontal brain lobe impairment in obstructive sleep apnoea: a proton MR spectroscopy study. *European Respiratory Journal*.

[30] Algin O., Gokalp G., Ocakoglu G., Ursavas A., Taskapilioglu O., Hakyemez B. (2012). Neurochemical–structural changes evaluation of brain in patients with obstructive sleep apnea syndrome. *European Journal of Radiology*.

[31] Öztürk S. B., Öztürk A. B., Soker G., Parlak M. (2018). Evaluation of brain volume changes by magnetic resonance imaging in obstructive sleep apnea syndrome. *Nigerian Journal of Clinical Practice*.

[32] Bartlett D. J., Rae C., Thompson C. H. (2004). Hippocampal area metabolites relate to severity and cognitive function in obstructive sleep apnea. *Sleep Medicine*.

[33] Morrell M. J., Jackson M. L., Twigg G. L. (2010). Changes in brain morphology in patients with obstructive sleep apnea. *Thorax*.

[34] Joo E. Y., Tae W. S., Lee M. J. (2010). Reduced brain gray matter concentration in patients with obstructive sleep apnea syndrome. *Sleep*.

[35] Torelli F., Moscufo N., Garreffa G. (2011). Cognitive profile and brain morphological changes in obstructive sleep apnea. *NeuroImage*.

[36] Sparks D. L., Kryscio R. J., Connor D. J. (2010). Cholesterol and cognitive performance in normal controls and the influence of elective statin use after conversion to mild cognitive impairment: results in a clinical trial cohort. *Neurodegenerative Diseases*.

[37] Rademeyer M., Joubert P. (2016). A comparison between the mini-mental state examination and the montreal cognitive assessment test in schizophrenia. *South African Journal of Psychiatry*.

[38] Siqueira G. S. A., Hagemann P. M. S., Coelho D. S., Santos F. H. D., Bertolucci P. H. F. (2019). Can MoCA and MMSE be interchangeable cognitive screening tools? a systematic review. *The Gerontologist*.

[39] An Y., Zhang X., Wang Y. (2019). Longitudinal and nonlinear relations of dietary and Serum cholesterol in midlife with cognitive decline: results from EMCOA study. *Molecular Neurodegeneration*.

[40] Formiga F., Ferrer A., Chivite D., Pinto X., Cuerpo S., Pujol R. (2011). Serum high-density lipoprotein cholesterol levels, their relationship with baseline functional and cognitive status, and their utility in predicting mortality in nonagenarians. *Geriatrics and Gerontology International*.

[41] Zeng F., Wang X., Hu W., Wang L. (2017). Association of adiponectin level and obstructive sleep apnea prevalence in obese subjects. *Medicine*.

[42] Lu M., Fang F., Wang Z., Wei P., Hu C., Wei Y. (2019). Association between serum/plasma levels of adiponectin and obstructive sleep apnea hypopnea syndrome: a meta-analysis. *Lipids in Health and Disease*.

[43] Chirinos D. A., Gurubhagavatula I., Broderick P. (2017). Depressive symptoms in patients with obstructive sleep apnea: biological mechanistic pathways. *Journal of Behavioral Medicine*.

[44] Petta S., Tuttolomondo A., Gagliardo C. (2016). The presence of white matter lesions is associated with the fibrosis severity ofnonalcoholic fatty liver disease. *Medicine*.

[45] Berger S., Polotsky V. Y. (2018). Leptin and leptin resistance in the pathogenesis of obstructive sleep apnea: a possible link to oxidative stress and cardiovascular complications. *Oxidative Medicine and Cellular Longevity*.

[46] Warren M. W., Hynan L. S., Weiner M. F. (2012). Leptin and cognition. *Dementia and Geriatric Cognitive Disorders*.

[47] Lal C., Strange C., Bachman D. (2012). Neurocognitive impairment in obstructive sleep apnea. *Chest*.

[48] Zuurbier L. A., Vernooij M. W., Luik A. I. (2016). Apnea-hypopnea index, nocturnal arousals, oxygen desaturation and structural brain changes: a population-based study. *Neurobiol Sleep Circadian Rhythms*.

[49] Filley C. M. (2019). History of subcortical cognitive impairment. *Frontiers of Neurology and Neuroscience*.

[50] Zhang X., Ma L., Li S., Wang Y., Wang L. (2011). A functional MRI evaluation of frontal dysfunction in patients with severe obstructive sleep apnea. *Sleep Medicine*.

[51] Parikh M. P., Gupta N. M., McCullough A. J. (2019). Obstructive sleep apnea and the liver. *Clinics in Liver Disease*.

[52] Ng S. S. S., Wong V. W. S., Wong G. L. H. (2021). Continuous positive airway pressure does not improve nonalcoholic fatty liver disease in patients with obstructive sleep apnea. a randomized clinical trial. *American Journal of Respiratory and Critical Care Medicine*.

